# An Iterative 3D Correction plus 2D Inversion Procedure to Remove 3D Effects from 2D ERT Data along Embankments

**DOI:** 10.3390/s24123759

**Published:** 2024-06-09

**Authors:** Azadeh Hojat

**Affiliations:** 1 Dipartimento di Ingegneria Civile e Ambientale, Politecnico di Milano, 20133 Milan, Italy; azadeh.hojat@polimi.it; 2 Department of Mining Engineering, Shahid Bahonar University of Kerman, Kerman 76188, Iran

**Keywords:** dam, earthen embankment, river levee, electrical resistivity tomography, 2D inversion, 2D/3D forward modelling, 3D effects

## Abstract

This paper addresses the problem of removing 3D effects as one of the most challenging problems related to 2D electrical resistivity tomography (ERT) monitoring of embankment structures. When processing 2D ERT monitoring data measured along linear profiles, it is fundamental to estimate and correct the distortions introduced by the non-uniform 3D geometry of the embankment. Here, I adopt an iterative 3D correction plus 2D inversion procedure to correct the 3D effects and I test the validity of the proposed algorithm using both synthetic and real data. The modelled embankment is inspired by a critical section of the Parma River levee in Colorno (PR), Italy, where a permanent ERT monitoring system has been in operation since November 2018. For each model of the embankment, reference synthetic data were produced in Res2dmod and Res3dmod for the corresponding 2D and 3D models. Using the reference synthetic data, reference 3D effects were calculated to be compared with 3D effects estimated by the proposed algorithm at each iteration. The results of the synthetic tests showed that even in the absence of a priori information, the proposed algorithm for correcting 3D effects converges rapidly to ideal corrections. Having validated the proposed algorithm through synthetic tests, the method was applied to the ERT monitoring data in the study site to remove 3D effects. Two real datasets from the study site, taken after dry and rainy periods, are discussed here. The results showed that 3D effects cause about ±50% changes in the inverted resistivity images for both periods. This is a critical artifact considering that the final objective of ERT monitoring data for such studies is to produce water content maps to be integrated in alarm systems for hydrogeological risk mitigation. The proposed algorithm to remove 3D effects is thus a rapid and validated solution to satisfy near-real-time data processing and to produce reliable results.

## 1. Introduction

In recent decades, there has been an increasing interest in integrating geophysical monitoring techniques with other technologies to assess the stability of slopes and embankments to mitigate hydrogeological risks [1,2]. Most long-term monitoring systems have been developed using microseismic networks to detect the seismic energy released by unstable slopes [3,4,5,6] or geoelectrical methods to monitor the hydrogeological conditions of slopes and embankments [7,8,9,10,11,12,13]. Among different geophysical techniques, electrical resistivity tomography (ERT) method has a long history in solving a variety of engineering, environmental and hydrogeological problems [14,15,16]. The most common applications include detection of different types of subsurface voids and cavities [17,18,19,20], mineral exploration [21,22], characterization of landfills, mining dumps and heap leaching facilities [23,24,25,26,27], and groundwater studies [28,29]. In recent years, the ERT method has continuously proven itself as an efficient element to be integrated in hydrogeological risk mitigation strategies, thanks to its potential to monitor changes in water saturation of the subsurface material and to detect seepage zones [30,31,32,33,34,35,36,37,38,39,40,41].

**Figure 1:**
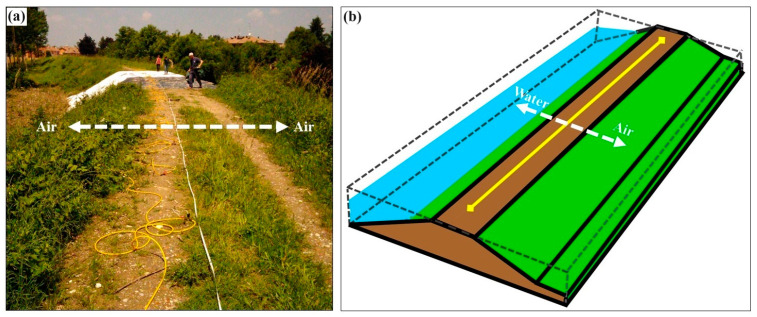
The special 3D geometry of earthen embankments that results in significant resistivity changes in the direction (white arrow) orthogonal to 2D ERT profile along the embankment (yellow cable/line). (**a**) The repaired river levee of the study site. (**b**) The schematic illustration of the river levee in a general case.

This paper specifically addresses ERT monitoring of earthen embankments like river levees, earthen dams, tailings dams and transportation embankments [1,11,12,13,32,39]. Two-dimensional ERT monitoring systems can be installed along the main axis of such structures with the aim of providing real-time measurements to highlight the internal heterogeneities of the embankments and, if required, activate early-warning alarm systems to promptly plan mitigation actions. The data acquired along an ERT line is a 2D pseudosection of apparent resistivity values which then undergoes an inversion procedure to produce a tomographic image that represents the real resistivity distribution of the embankment body along the ERT line and with depth. A 2D ERT inversion algorithm assumes that the resistivity does not change in the direction perpendicular to the ERT profile, but the special 3D geometry of earthen embankments (Figure 1) does not satisfy this requirement and thus, distortions are introduced into the 2D data measured along these structures [42,43,44,45,46,47]. Such distortions are known as 3D effects and depend on the specific 3D geometry of each site, the resistivity distribution within the embankment, and the boundary conditions of the embankment (i.e., air, water or a combination of air and water on either side).

It is thus necessary to quantify the site-specific 3D effects for 2D ERT profiles measured along embankments. Hojat et al. (2020) performed numerical modelling and laboratory measurements on downscaled levees and after validating their method by laboratory experiments, they calculated the seasonal 3D effects for a real site. Their results showed the importance of removing 3D effects to have reliable results. Moreover, it was observed that 3D effects are maximum when air is present on both sides of the embankment and they continue to be reduced as the water level in the river side is increased [47]. Synthetic modelling was followed by Ball et al. (2023) for a homogeneous and heterogeneous embankment to simulate the varying water levels and salinities to explore 3D artefacts. Their results confirmed again that 3D effects are a function of the embankment geometry, geology and water content as well as boundary conditions [46]. Thus, the problem is further emphasized knowing that 3D effects not only depend on the geometry of the embankment, but they are also affected by seasonal variations in the resistivity values in the embankment body as well as by the fluctuations of the air/water levels in either side of the embankment [46,47]. Zanzi and Hojat (2023) proposed an iterative 3D correction plus 2D inversion procedure to correct 2D ERT data for 3D effects and then invert the corrected data using a 2D inversion algorithm [43]. In this paper, I discuss a couple of examples from the various synthetic modeling studies performed to validate the method. I also present two examples from long-term monitoring data measured along a river levee after rainy and dry periods to underline the importance of 3D effects. We have different projects where customized ERT monitoring systems are permanently installed along a river levee or a tailings dam—e.g., [13,47,48]. In most projects, the earthen embankment has an approximately homogeneous body and the main inhomogeneity that can be considered for resistivity values is a layered situation due to rainy or dry seasons that result in a decrease or increase in the resistivity values of the superficial part of the embankment. One exception pilot site in our projects is a critical part of the Parma River levee where lateral changes are also present in the levee body. I have therefore focused on this study site for one of the synthetic tests discussed in this paper.

## 2. The Study Site

**Figure 2:**
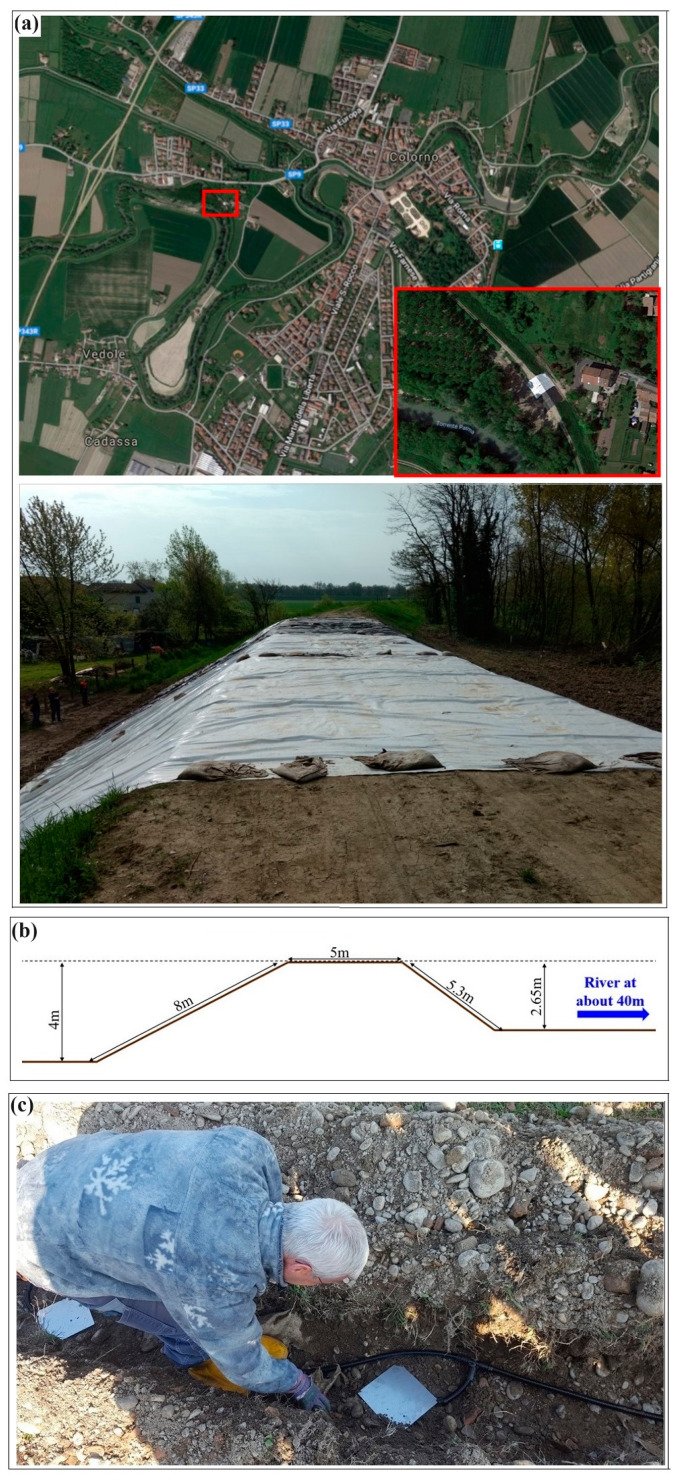
(**a**) The study site in Colorno, La Penza, Italy. The red rectangles on the top picture show the critical part of the river levee that had experienced a water leakage and was repaired. The picture on the bottom shows a better view of the reconstructed section where the monitoring system was later installed. (**b**) The cross-section of the river levee looking at the picture shown on the bottom in (**a**). (**c**) Permanent installation of the ERT cables and plate electrodes inside a 0.5 m deep trench.

The study site is located near Colorno city in central Italy and had experienced a water loss event in a part of the Parma River levee in 2017. Although the defective section was immediately repaired (Figure 1a and Figure 2a), the necessity of permanently monitoring the critical section that faces a farm and transportation infrastructure downstream was strengthened. Numerous preliminary measurements were performed along candidate sites in the zone and the same section of the Parma River levee was finally selected for the permanent installation of a customized ERT monitoring system [48]. The cross-section of the river levee orthogonal to the ERT line is shown in Figure 2b. The Parma River is located about 40 m from the monitored section of the levee in the study site. Air is often present on both sides of the river levee with the exception of periods of heavy rainfall when the water might come into contact with the levee. The autonomous ERT system that is composed of two 24-channel cables and 48 plate electrodes (20 × 20 cm) was installed in November 2018. The protected cables and the electrodes were buried in a 0.5 m deep trench (Figure 2c). Based on the results obtained during the reconnaissance studies, the Wenner electrode configuration with the unit electrode spacing of 2 m was selected for permanent data acquisitions. Readers interested in more information about the installation details in the study site are referred to Hojat et al. (2019) [48].

## 3. Correcting 3D Effects

**Figure 3:**
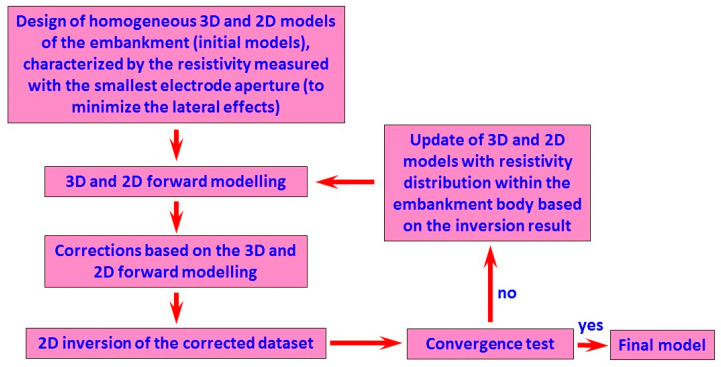
Flow chart of the proposed iterative procedure to apply corrections of 3D effects.

Three-dimensional inversion would be the solution to take into account the 3D effects of the embankment structures during data processing. However, since 2D acquisition systems are the most commonly used systems along such structures because of convenient field work, adequate coverage of subsurface anomalous zones, and relatively rapid data processing and real-time interpretation [46,47], a 3D inversion would be very poorly constrained. Therefore, an iterative 3D correction + 2D inversion procedure was proposed [43]. The proposed iterative procedure is described in Figure 3 and by Equations (1) and (2). The measured apparent resistivity data ($\rho_{a}^{M}$) should be divided by the amplification caused by the 3D effects. In the case of having adequate knowledge about the resistivity distribution within the embankment, no iterative procedure is required and 3D effects can be immediately estimated. To achieve this, 2D and 3D synthetic models of the embankment are defined and synthetic data are calculated for these models. The amplification factor (α) is obtained by dividing the synthetic data calculated for the 3D model (containing 3D effects) by the synthetic data calculated for the 2D model (free from 3D effects). Normally, the resistivity distribution within the embankment is not known and the iterative algorithm described here can solve the problem. The procedure starts with an initial assumption about resistivity distribution within the embankment. In the absence of any a priori information, a homogenous resistivity distribution is considered with the resistivity value defined as the average of the data measured by the smallest electrode spacing because 3D effects are minimal for the shallowest data. The 2D and 3D synthetic models are then defined and 3D effects are estimated for the assumed initial models. The measured data are then corrected for 3D effects and inverted using a 2D inversion algorithm. The synthetic 2D and 3D models are now updated using the resistivity distribution obtained from the inverted model. The 2D and 3D synthetic data are calculated again on the updated models and the 3D effects are estimated for the new iteration. The measured data are corrected using the updated 3D effects and the corrected data are inverted. The iteration procedure is repeated, updating the synthetic 2D and 3D models using the resistivity distribution obtained from the new inverted model and so on. The procedure stops when the convergence test is satisfied and negligible changes are observed between two successive iterations. As I will show in the following sections, one or two iterations are normally enough to arrive at accurate estimations of the 3D effects.

The correction procedure is also explained by Equations (1) and (2). The data corrected for the 3D effects ($\rho_{a}^{c_{j}}$) at each iteration (j) are obtained by dividing the measured data ($\rho_{a}^{M})$ by the correction factors ${(\alpha }_{j}$), Equation 1. The correction factor at each iteration is calculated as the ratio of the apparent resistivities simulated on the 3D model ($\rho_{a}^{{3D}_{j}}$) to the apparent resistivities simulated on the corresponding 2D model ($\rho_{a}^{{2D}_{j}}$) at the same iteration, Equation (2).

(1) $$\rho_{a}^{c_{j}}=\frac{\rho_{a}^{M}}{\alpha_{j}}$$

with

(2) $$\alpha_{j}=\frac{\rho_{a}^{{3D}_{j}}}{\rho_{a}^{{2D}_{j}}}$$

Note that 3D models should be defined using the knowledge of the embankment geometry as well as the boundary conditions. The geometry of earthen embankments like dams and tailings dams is normally known. In any case, drones can be used to accurately reconstruct the morphology of the monitored embankment. As far as the boundary conditions are concerned, i.e., the presence and the level of air and/or water on either side, ERT monitoring systems are usually accompanied by meteorological stations and water level sensors.

In order to validate the proposed correction strategy, the method was first tested on several synthetic models. Then the proposed correction procedure was used to process the ERT monitoring data obtained in a few of our projects where ERT monitoring systems are installed along earthen embankments. In this paper, I illustrate a couple of synthetic tests along with some selected field data from the Colorno study site.

### 3.1. Synthetic Models

Various synthetic simulations were performed with and without the presence of water on the river side and defining different water levels. The two examples selected to be discussed in this paper consider only air on both sides of the river levee because 3D effects were reduced by the presence of water in the river side. This is due to the fact that in our tests (inspired by our monitoring sites in fresh environments), the conductivity of the river water is not too high to generate an exaggerated contrast with the levee material. However, it should be mentioned that 3D effects are enhanced in the brackish waters common in tidal environments [46]. The other reason for selecting the simulations when only air is present on both sides of the embankment is that, as described in Section 2 (see also Figure 2b), Parma River passes at a distance of about 40 m from the ERT line. This distance is far enough to ignore the 3D effects caused by the river body [46]. Therefore, the tests selected for this discussion are:

1. Synthetic test no. 1 presents one of the most common situations for a more-or-less homogeneous embankment resting on a conductive clayey subsoil. This model also considers a superficial resistive layer that covers river levees (like a gravel road for cyclists and runners).
2. Synthetic test no. 2, on the other hand, presents simulations inspired by the special composition of the study site where there is a central inhomogeneous zone due to a repair operation.

I used Res2dmod (ver. 3.03.06) and Res3dmod (ver. 2.14.23) software to define 2D and 3D synthetic models and perform forward calculations to generate synthetic data (www.geotomosoft.com, accessed on 6 June 2024). Synthetic data were generated for the Wenner electrode configuration using 30 electrodes with unit electrode spacing of 2 m. In this study, I have used Res2dinvx64 (ver. 5.0) software (Copyright © Seequent Systems, Incorporated) for 2D inversions. In the following sections, I always use the central profile along the main axis of the embankment from the synthetic data calculated in Res3dmod.

#### 3.1.1. Synthetic Test No. 1

**Figure 4:**
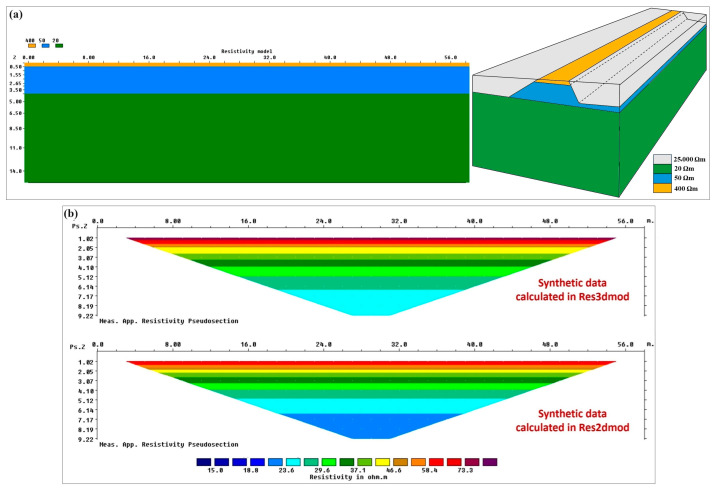
Synthetic test no. 1: (**a**) the reference 3-layered 2D model (**left**) and the corresponding 3D model obtained by extrapolating laterally the 2D model and assuming the presence of air on both sides (**right**). (**b**) Synthetic data calculated over 3D model (**top**) and 2D model (**bottom**).

The first reference model was defined as a simplified three-layered model for the 2D section cut along the longitudinal axis of the embankment (Figure 4a, left). The first layer of the 2D vertical model is the typical unpaved road that often runs along river levees. This superficial gravel layer is modelled by a 0.5 m thick layer with the resistivity of 400 Ωm. Below the superficial gravel rests a homogeneous embankment with the resistivity of 50 Ωm that extends down to 4 m. The river levee is assumed to be on a homogeneous subsoil with the resistivity of 20 Ωm. The corresponding reference 3D model (Figure 4a, right) is obtained by extrapolating laterally the 2D model according to the cross-section geometry illustrated in Figure 2b, and assuming the presence of air with the resistivity of 25,000 Ωm on both sides of the levee. The 3D model shown in Figure 4a, right, is only a schematic illustration and is not precisely scaled. I used the 3D sensitivity plot for the Wenner array to decide the 3D extension of the embankment [49]. The area with significant sensitivity extends to approximately 0.5 times the array length from the first and the last electrodes in the x direction (along the ERT profile) and about 0.5–0.7 times the array length in the y direction (the off-axis direction).

The defined 2D and 3D reference models were used for calculating the reference 3D effects for this synthetic test. First, the synthetic data were calculated for the 2D model and 3D model in Res2dmod and Res3dmod, respectively. The calculated synthetic data are shown in Figure 4b. The 3D model in Res3dmod considers the 3D structure of the embankment and its boundary conditions (air on both sides). Therefore, the data calculated in Res3dmod will actually represent the raw measured data, i.e., the data distorted by the 3D geometry of the embankment. The 2D model in Res2dmod, on the other hand, assumes that the resistivity changes only in two directions, i.e., along the ERT profile and with depth. Therefore, the data calculated in Res2dmod will not reflect 3D effects and they actually represent the desired measured data, i.e., the measured data after being fully corrected for 3D effects. A comparison of the synthetic data illustrated in Figure 4b clearly shows that the measured data in a real case are distorted by 3D effects.

**Figure 5:**
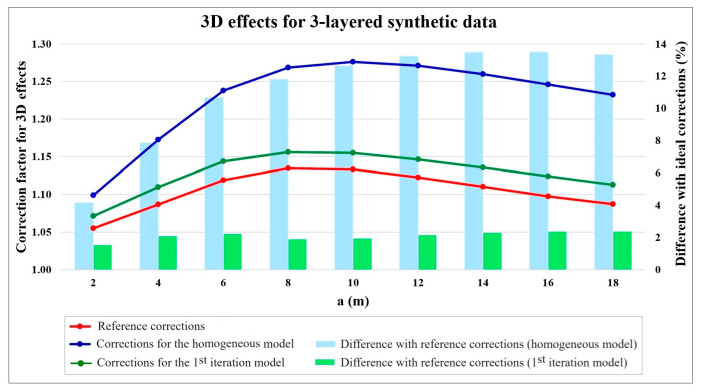
Synthetic test no. 1: correction factors as a function of electrode spacing, a, for the reference model (red graph) and for different models of the proposed iterative procedure.

Using the defined reference 2D and 3D models, I calculated the reference 3D effects for comparison with different steps of the proposed iterative correction procedure. The reference 3D effects are presented as the red graph in Figure 5. This graph was obtained by dividing the synthetic data calculated on the 3D model by the synthetic data calculated on the 2D model. The 3D effects can be presented as graphs plotted versus electrode spacing for test no. 1 because 3D effects vary only with penetration depth in the absence of lateral inhomogeneities.

**Figure 6:**
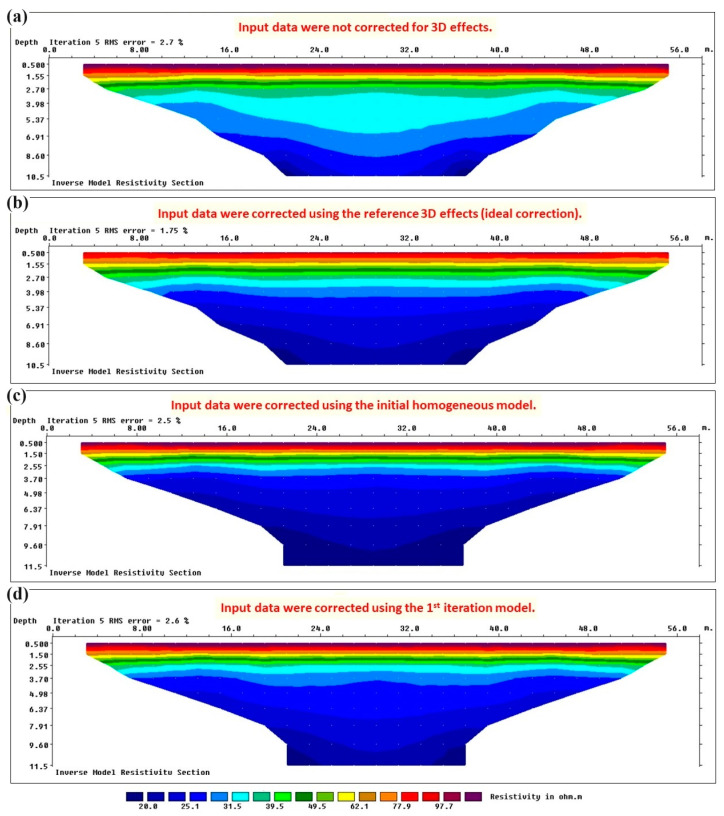
Inverted resistivity sections for synthetic “real” data of test no. 1. (**a**) Without removing 3D effects. (**b**) The 3D effects were removed using ideal corrections. (**c**) The 3D effects were removed using the initial homogeneous model of the iterative correction method. (**d**) The 3D effects were removed using the first iteration model of the iterative correction method.

The red graph in Figure 5 shows that a maximum amplification factor of 1.135 distorts the measured apparent resistivity data for synthetic test no. 1. In order to highlight the importance of estimating and removing such 3D effects from the measured data, I demonstrate the resistivity sections obtained after inverting the synthetic data calculated in Res3dmod without applying 3D corrections (Figure 6a) and after applying reference 3D-effect corrections (Figure 6b). As mentioned, the data calculated in Res3dmod simulate the “real” data measured in the field because they are distorted by the 3D geometry of the embankment, i.e., they not only reflect resistivity changes along the ERT profile and with depth, but they are also affected by lateral 3D effects. Since I have considered air on both sides of the embankment, 3D effects have amplified the measured apparent resistivity values (Figure 4b, top). If the data are not corrected for 3D effects, unreal resistivity values are obtained in the inverted section (Figure 6a) compared to the real resistivity distribution (Figure 6b). While in Figure 6b, the inverted resistivity at the base of the levee (4 m) rapidly converges towards about 20 Ωm that is the correct value of the subsoil, in Figure 6a, the decrease in the inverted resistivity with depth is remarkably delayed and the 20 Ωm value is approached only around the depth of 10 m. Note that in this test, Figure 6b represents the inverted reference section, i.e., the desired inversion result obtained from the synthetic data calculated on the corresponding 3D model (Figure 4b, top) and inverted after being corrected for 3D effects using the red graph in Figure 5.

Now the proposed iterative procedure can be tested to correct 3D effects on “real” data (i.e., synthetic data calculated on the reference 3D model). I assume that no a priori information is available about the internal structure of the embankment body. Thus, I have to start from the simplest possible initial model which is a homogeneous model. Assuming that 3D effects are negligible for the first depth level of measurements (about 1 m) on a 5 m wide levee, I used the average value of the apparent resistivity data acquired at the shallowest parts along the profile (about 70 Ωm) to define the resistivity value for the homogeneous model. Using 2D and 3D synthetic data calculated on this homogeneous embankment, 3D corrections were obtained (blue graph in Figure 5). It should be noted that for the initial homogeneous 2D model, there was no need to run the forward modelling software because the result is expected to be 70 Ωm for all the data points. Having calculated the 3D effects for the initial homogeneous model, the “real” data were submitted to the first corrections for 3D effects and were then inverted in Res2dinvx64 software. The inverted section is shown in Figure 6c. This section was used to modify the initial homogenous model for the next iteration. The modified model (named the first iteration model) consists of a 1.5 m thick superficial layer with the resistivity of 80 Ωm over a homogeneous embankment with the resistivity of 40 Ωm down to 5 m. The third layer is the subsoil with the resistivity of 25 Ωm. The corresponding 2D and 3D models were defined in Res2dmod and Res3dmod, respectively, and synthetic data were calculated. The 3D effects were then recalculated for this first iteration model (green graph in Figure 5) and corrections were applied again to the reference “real” data. The new inverted section is shown in Figure 6d and the rapid convergence of the results can be immediately noted comparing Figure 6d with the reference section shown in Figure 6b or comparing the green graph with the reference red graph in Figure 5.

#### 3.1.2. Synthetic Test No. 2

**Figure 7:**
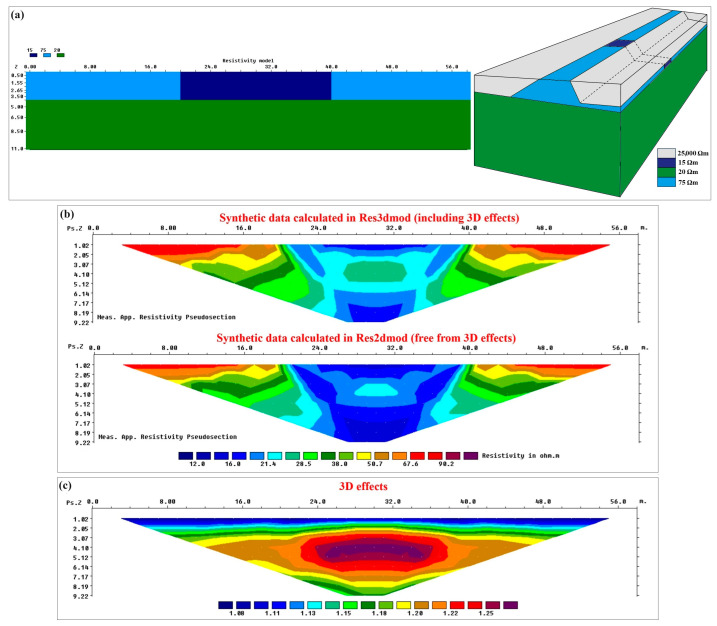
Synthetic test no. 2: (**a**) the reference 2D synthetic model for a laterally inhomogeneous embankment (**left**) and the corresponding 3D model obtained by extrapolating laterally the 2D model and assuming the presence of air on both sides (**right**). (**b**) Synthetic data calculated over 3D model (**top**) and 2D model (**bottom**). (**c**) The 3D effects.

The second example to validate the proposed strategy to remove 3D effects is based on the special laterally inhomogeneous structure of the river levee in the study site. The reference 2D model is illustrated in Figure 7a, left, and considers an embankment with the resistivity of 15 Ωm in the central part and the resistivity of 75 Ωm in the lateral zones. This is compatible with the fact that the central part of the study site was repaired with clayey material after experiencing a seepage event. The embankment height is assumed to be 4 m resting on a homogeneous subsoil with the resistivity of 20 Ωm. The corresponding 3D model was obtained by extrapolating laterally the 2D model and assuming the presence of air on both sides (Figure 7a, right). Similar to synthetic test no. 1, the geometry of the levee is reconstructed using the cross-section geometry illustrated in Figure 2b. However, the 3D model shown in Figure 7a is only a schematic illustration and is not precisely scaled.

**Figure 8:**
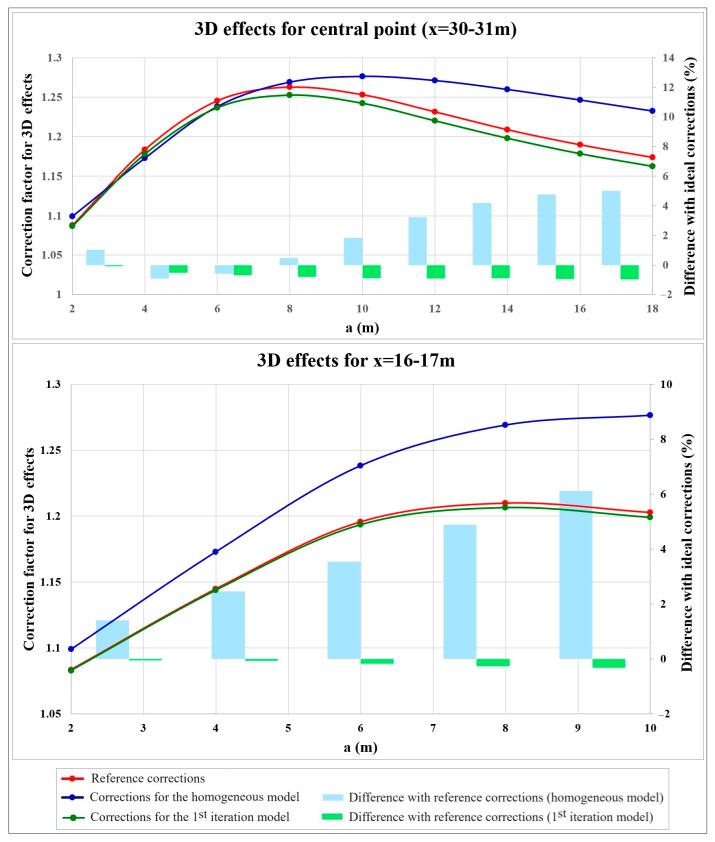
Correction factors as a function of electrode spacing, a, for synthetic test no. 2 for the central part (x = 30–31 m, **top**) and the lateral zones (x = 16–17 m, **bottom**).

Similar to all synthetic tests, the reference 2D and 3D models of test no. 2 were used to calculate the reference (ideal) 3D effects. To do this, synthetic apparent resistivity pseudosections on the 3D and 2D models were respectively calculated in Res3dmod and Res2dmod (Figure 7b). It can be observed, in Figure 7b, how the measured apparent resistivity values are amplified in the presence of 3D effects for the data calculated in Res3dmod. Reference 3D effects were then obtained by dividing the data containing 3D effects (calculated on the 3D model, Figure 7b, top) by the data without 3D effects (calculated on the 2D model, Figure 7b, bottom). The reference 3D effects for synthetic test no. 2 are shown in Figure 7c and it can be observed that the 3D effects do not vary only with depth but also laterally. The 3D effects are more severe for the central part that has a higher resistivity contrast with air that is the material present in the 3D sides of the embankment. However, considering the symmetric structure of the embankment and of the 3D effects, two reference graphs could be extracted for the 3D effects; one for the lateral zones and one for the central part of the embankment (red curves in Figure 8). It should be noted that the graphs presented in Figure 8 are aimed to compare the 3D effects for the two different zones of the embankment modelled in synthetic test no. 2. The 3D geometry and boundary conditions of the embankment are similar for both graphs and the only difference is the resistivity distribution within the levee for the considered zones. Of course, I used the complete section of 3D effects for the iterative procedure to correct the data for test no. 2.

**Figure 9:**
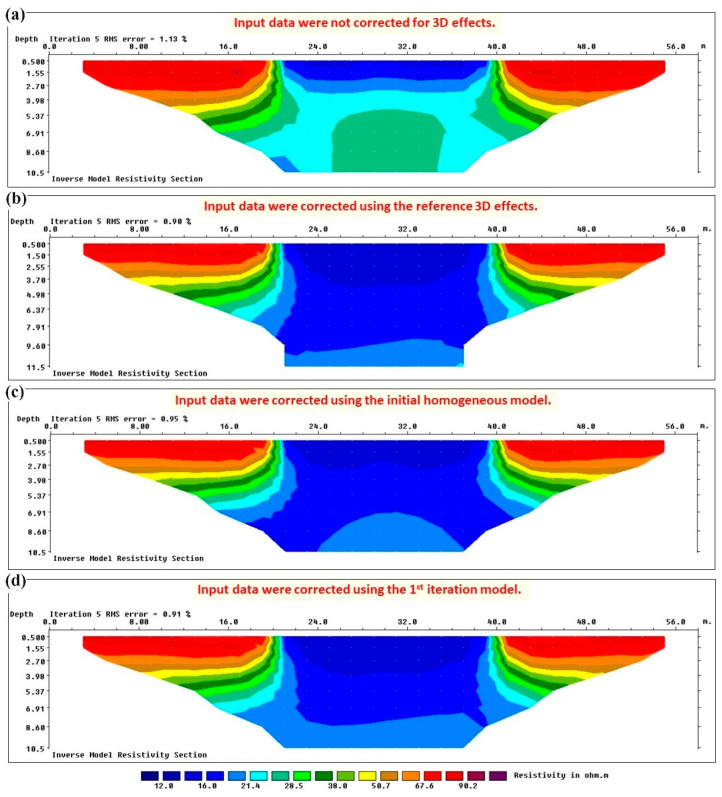
Inverted resistivity sections for synthetic “real” data of test no. 2. (**a**) Without removing 3D effects. (**b**) The 3D effects were removed using ideal corrections. (**c**) The 3D effects were removed using the initial homogeneous model of the iterative correction method. (**d**) The 3D effects were removed using the first iteration model of the iterative correction method.

Figure 9a demonstrates the resistivity section obtained after inverting the synthetic data calculated in Res3dmod without applying 3D corrections, while Figure 9b illustrates the reference inverted resistivity section obtained from 3D-effect-free data, i.e., the synthetic data calculated in Res2dmod over the reference 2D model or the synthetic data calculated in Res3dmod over the reference 3D model after applying the reference (ideal) 3D-effect corrections. The comparison shows the importance of correcting 3D effects before inversion, as was already observed by comparing Figure 6a with Figure 6b for synthetic test no. 1. Again, inverted resistivities obtained without the corrections are largely overestimated and this would produce a remarkable underestimation of the water content of the structure.

Then, similar to the previous example, I tested the proposed iterative procedure to correct 3D effects on reference “real” data (i.e., synthetic data calculated on the reference 3D model). Again, I assume to start without a priori information and thus, I defined the simplest initial model. Although from raw apparent resistivity data, I can have the idea of a laterally inhomogeneous structure, in order to test the convergence of the correction algorithm, I assumed a homogeneous model with the resistivity value of 50 Ωm selected as the average value of the data measured at the shallowest parts along the profile. After calculating the 3D effects for this initial homogeneous model (blue graphs in Figure 8), the “real” data were corrected for 3D effects and were then inverted in Res2dinvx64 software (Figure 9c). Using the inverted section of Figure 9c, the iterative procedure was followed by modifying the model to calculate 3D effects. The new model (named the first iteration model) is composed of a laterally inhomogeneous structure with the resistivity of 70 Ωm for the lateral parts and the resistivity of 16 Ωm for the central part located at x = 20–40 m. I also defined the more conductive subsoil with the resistivity of 19 Ωm. Having defined the modified 2D and 3D models, the 3D effects were recalculated (green graphs in Figure 8) and the reference “real” data were corrected. The inverted resistivity section for the data corrected at this iteration is shown in Figure 9d. I remind readers that the correction graphs illustrated in Figure 8 are two samples for the laterally different zones, but the complete section of 3D effects was used to correct the data at each iteration. Comparing the results shown in Figure 8 and Figure 9, the good convergence of the correction algorithm can be realized even for an embankment with significant lateral resistivity changes.

### 3.2. Field Data

In this section, I present two examples of the monitoring data from the study site measured after a rainy and a dry period. As mentioned in Section 2, the electrodes and the cables of the ERT monitoring system are buried in a 0.5 m deep trench at the study site. Since the equipment firmware calculates the apparent resistivity values using the standard geometrical factor for the Wenner configuration (2πa, a being the electrode spacing), the measured data are immediately corrected for the effect of the soil covering the electrodes [50] before applying 3D-effect corrections. It should be mentioned that when analyzing seasonal ERT data, the measured data are also corrected for soil temperature changes with depth. Annual soil temperature changes are calculated from the site-specific temperature model calibrated using the air temperature sensor and one or more soil temperature sensors installed in the study site. Details about temperature corrections are beyond the scope of this paper. In this section, I focus on two datasets measured after a dry and a rainy period without significant changes in soil temperature.

**Figure 10:**
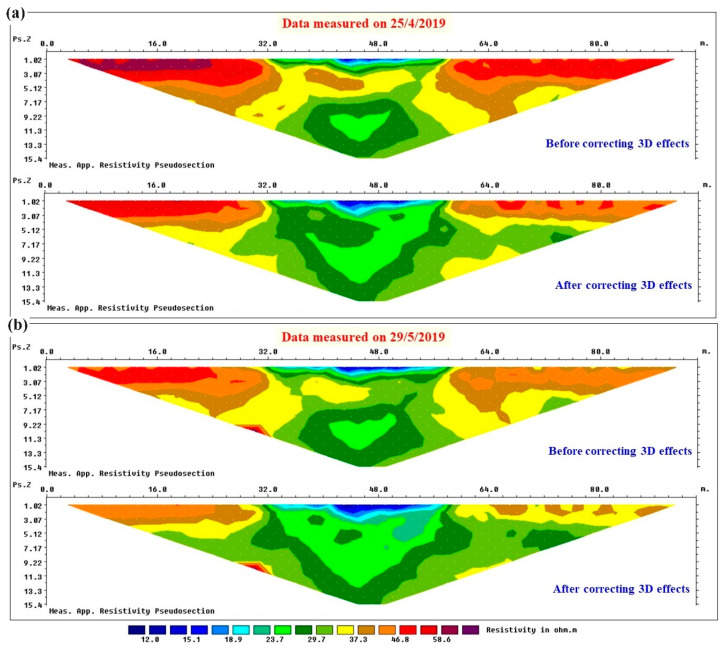
Examples of the data measured by the permanent ERT monitoring system in the study site before and after applying corrections for 3D effects. The data were measured on (**a**) 25 April 2019 after a dry week without rain, (**b**) 29 May 2019 after about one month of rain.

Figure 10a illustrates the apparent resistivity data measured on 25 April 2019 after a dry week without rain, while Figure 10b presents the apparent resistivity data measured on 29 May 2019 after about one month of rainfall in the study site. Each dataset is presented before and after applying the final corrections for 3D effects, obtained using the iterative procedure illustrated on the synthetic examples. In order to define synthetic 3D models for the correction procedure, the 3D geometry of the levee was obtained from drone measurements in the study site. Comparing each dataset in Figure 10 before and after applying 3D corrections, it can be realized that regardless of the absence or presence of rainfall and, accordingly, the different resistivity distributions in the levee, the measured apparent resistivity values were increased by 3D effects due to the presence of the air on both sides of the river levee for both dates. Moreover, the deepest parts of the measured apparent resistivity pseudosections at pseudodepths greater than about 9.5 m remained almost unchanged after applying the 3D corrections.

**Figure 11:**
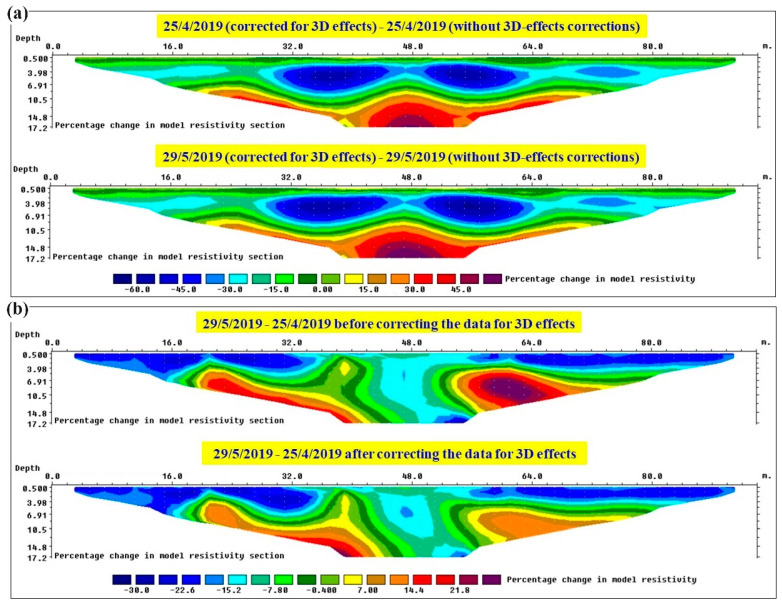
Percentage changes in inverted resistivity sections for the data measured on (**a**) 25 April 2019 (**top**) and 29 May 2019 (**bottom**) with and without 3D-effect corrections, (**b**) 29 May 2019 compared to 25 April 2019 before applying 3D-effect corrections (**top**) and after applying 3D-effect corrections (**bottom**).

The main objective of integrating ERT monitoring systems with hydrogeological risk mitigation strategies is to give early-warning alarms based on excessive water saturation distribution in the embankment body. Therefore, it is crucial to have the resistivity sections as accurate as possible in order to prepare reliable water saturation maps from the resistivity sections. Considering that 3D effects significantly distort ERT data measured along embankment structures, removing the 3D effects from the measured data is one of the important processing steps before inverting the data. In order to better highlight this important step, Figure 11 shows different images of the percentage changes in inverted resistivity sections for the example datasets presented in this section. The percentage changes in resistivity models for each individual section with and without 3D-effect corrections are shown in Figure 11a. Resistivity changes of about ±50% can be observed for both datasets when they are not corrected for 3D effects. This results in significant errors when transforming the resistivity sections into water saturation maps and, accordingly, misleading alarms might be launched. Figure 11b illustrates the percentage resistivity changes for the inverted resistivity sections obtained from the data measured on 29 May 2019 compared to the data measured on 25 April 2019 before and after removing 3D effects. Of course, observing the resistivity differences at two different times rather than the resistivity values at a single time attenuates the impact of the 3D effects. As a result, the two different maps obtained with and without 3D effects’ corrections seem qualitatively quite similar and both allow the user to understand which are the areas where the structure is more rapidly and heavily reacting to rainfall. Nevertheless. we can observe zones with non-negligible differences before and after corrections for 3D effects, which would result in remarkable quantitative misinterpretations about the variations in the water content of the structure.

In order to transform resistivity sections into water content maps, samples should be taken from the study site to calibrate the mathematical equation proposed by Waxman and Smits (1968) [51]. This procedure is supposed to be followed in the near future and we will publish the results in future studies on the long-term analysis of the ERT data at the study site.

## 4. Discussion

The synthetic simulations presented in this study highlight how 2D ERT data measured along embankment structures are significantly influenced by resistivity changes in the direction perpendicular to the ERT profile, known as the 3D effects. This is due to the three-dimensional nature of current circulation in the subsurface. One general recommendation to account for 3D effects in ERT monitoring of embankment structures is to perform 3D ERT acquisitions and inversions because the 3D approach can incorporate the full embankment geometry as well as lateral resistivities [46]. However, several studies have shown that 2D ERT surveys deployed on the embankment crest can adequately satisfy the monitoring objective. The 2D approach combined with the proposed correction strategy thus has the advantage of removing 3D effects without the higher costs of 3D surveys and without the drawback of inversion instabilities due to the much higher level of ill-conditioning of the 3D problem compared to the 2D problem. Furthermore, although rapid developments in computer technology are continuously introduced, the 2D approach has the further benefit of reducing the required computational time and memory compared to 3D inversion, especially when the input to the inversion algorithm consists of a long sequence of resistivity maps that will be simultaneously inverted with a time-lapse inversion algorithm. The synthetic tests demonstrated that the algorithm to estimate 3D effects can rapidly converge to real values for vertically or laterally inhomogeneous embankments even in the absence of a priori information about the resistivity distribution in the embankment body. In both the synthetic and field data examples illustrated in this study, the presence of air on both sides of the river levee results in the amplification of the measured apparent resistivity values due to 3D effects. It can be observed in Figure 5, Figure 7c and Figure 8 that the 3D effects increase with the electrode spacing up to a maximum, after which, they start to decrease. The initial increasing trend of 3D effects with electrode spacing is due to the fact that while the electrode spacing is increased up to the spacing that investigates the full height of the embankment, lateral inhomogeneities present on the two sides of the embankment become progressively more and more important in affecting the 3D current flow. Considering a 4 m high levee as in the simulated and also the real examples discussed here and assuming an approximate penetration depth equal to one sixth of the spacing between the current electrodes, we can observe in Figure 5 and Figure 8 that maximum 3D effects occur for the electrode spacing a = 8 m. After this point, 3D effects continuously decrease with a further increase in electrode spacing (and accordingly, further penetration depth). The decrease in 3D effects is caused by the decreased percentage of the volume occupied by lateral inhomogeneities (air) on the two sides of the levee compared to the volume of the soil affected by current flow that is increasingly penetrating the subsoil.

A comparison of Figure 5 with Figure 8 shows that keeping the levee geometry and the boundary conditions unchanged, the 3D effects vary also with the resistivity distribution within the embankment body. Reference 3D effects for test no. 1 approach a maximum value of about 14% while the maximum 3D effects for test no. 2 are about 21% and 27% for the lateral and central zones, respectively. All these values are much larger than the normal measurement error of ±2% that is usually estimated in ERT measurements [1] and prove the importance of correcting the data for 3D effects. The variations in 3D effects with the resistivity distribution within the embankment body highlights the importance of defining reliable models to estimate 3D effects. However, synthetic test no. 2 demonstrated that despite starting the iterative procedure assuming an exaggeratedly simple homogeneous model, the iterative algorithm converged rapidly to ideal values with percentage differences less than 1% for all points (Figure 8).

Regarding the definition of the initial model, we might consider the fact that the construction materials are sometimes known for earthen embankments, especially for man-made embankments like dams and tailings dams, or like in this study, at least for the repaired zones. Moreover, talking about ERT monitoring projects for mitigating hydrogeological risks, the final demanded information to extract from resistivity images is often the water saturation. For this purpose, some coring operations are normally performed along the ERT line to calibrate the relationship between resistivity and water saturation. The coring material can also provide valuable information about the embankment material. Thus, some a priori information is often available to drive the definition of the initial model. In the absence of any possible a priori information, this work shows that a simple homogeneous initial model of the embankment body with a resistivity equal to the average value of the apparent resistivities measured with the smallest electrode spacing can be successfully used to start the iterative procedure. As a matter of fact, all the synthetic tests rapidly converged after only one iteration to reference 3D effects with percentage differences less than 2%.

Having obtained promising results, the proposed procedure to remove 3D effects is being applied to datasets measured by the ERT monitoring system at the study site (and also to those at our other embankment-monitoring projects). As an example, I selected two datasets for two different periods: after some dry and after some rainy weeks. A comparison of the measured apparent resistivity pseudosections before and after removing the 3D effects (Figure 10) for each dataset shows that the observed trend of 3D effects with depth (i.e., with electrode spacing) is also valid for the real data. For pseudodepths approximately equal to the levee height (i.e., 4 m), 3D effects arrive at the maximum values (i.e., the highest differences are observed between the pseudosections before and after corrections). The differences start decreasing for larger pseudodepths and at about 9–9.5 m, the apparent resistivities before and after corrections converge to very similar values because the 3D effects become negligible at greater depths. A comparison of the inverted sections for the data with and without 3D effects showed that the presence of 3D effects for the specific geometry of the studied river levee and with air on both sides of the levee results in artifacts in the order of ±50% (Figure 11a). This can significantly affect the water-content maps calculated from the resistivity images and can result in the unreliable definition of alarming algorithms.

It is worth mentioning that Ball et al. (2023) compared the 3D effects for the Wenner, Schlumberger and the dipole–dipole electrode configurations. Their results demonstrated the highest sensitivity of the dipole–dipole electrode configuration to off-line resistivity variations, while the Wenner electrode array is less likely to be influenced by 3D effects [46]. Although less sensitive, this study demonstrates that the data measured with the Wenner array on dams or levees cannot be quantitatively interpreted by ignoring the 3D effects. This issue is more critical when other common electrode configurations are used for data acquisition, particularly the dipole–dipole configuration.

## 5. Conclusions

The results of different synthetic tests demonstrated that the proposed iterative 3D correction plus 2D inversion procedure can rapidly converge to ideal 3D corrections. The tests explored the convergence of the iterative algorithm assuming a complete absence of a priori information about the embankment material, but usually some information might be available from construction documents or coring operations at each site. Using such information, a model closer to reality can be defined resulting in an even more rapid and accurate estimation of 3D effects, normally after the first iteration. The results also showed that 3D effects not only vary with the 3D geometry and the boundary conditions of the embankment, but they are also affected by the resistivity distribution within the embankment. For a homogenous river levee with a resistive superficial layer, resistivity values were increased by 14% while for a laterally inhomogeneous river levee, resistivity values were increased by 21% and 27% for the lateral zones and the central part, respectively. In all these tests, maximum 3D effects occurred at the electrode spacing equivalent to the embankment height. Laterally different 3D effects due to the lateral inhomogeneity of the river levee were also observed in the data from the study site. Larger artifacts occurred in the central part of the ERT line where the levee was repaired with a more conductive clayey material. One main advantage of the proposed approach, consisting of iterative 3D corrections plus 2D inversions, is that the computational time and memory required by a full 3D inversion is considerably decreased. This is a critical issue when dealing with ERT data for real-time monitoring purposes where a few datasets are daily acquired to launch alarms in case of excessive water saturation events. A second important advantage of the proposed approach is that the 2D inversion problem is much better constrained than the 3D inversion problem and thus the final solution is much more reliable and stable.

## Data Availability

Datasets used in this research will be sent to interested researchers upon request.
